# Gatekeeping Dietary Fiber: The Role of Carbohydrate-Binding Modules in the Human Gut

**DOI:** 10.4014/jmb.2601.01042

**Published:** 2026-03-16

**Authors:** Inonge Noni Siziya, Cheon-Seok Park, Dong-Hyun Jung

**Affiliations:** 1 Division of Food and Nutrition, Chonnam National University, Gwangju 61186, Republic of Korea; 2 Department of Food Science and Biotechnology, Graduate School of Biotechnology and Institute of Life Science and Resources, Kyung Hee University, Yongin 17104, Republic of Korea

**Keywords:** Carbohydrate-binding modules, Gastrointestinal tract ecology, Microbial fiber utilization, Polysaccharide metabolism

## Abstract

Carbohydrate-binding modules (CBMs) are widely recognized as accessory domains that enhance polysaccharide hydrolysis by carbohydrate-active enzymes. Using genomic surveys and ecological mapping across the human gastrointestinal tract, we outline three previously unrecognized principles of CBM distribution. First, CBM repertoires show substrate-axis specialization consistent with the carbohydrate targets of different microbial groups. Second, patterning emerges along the oral–ileal–colonic axis, reflecting spatial gradients in substrate availability and microbial niches. Third, CBM compositions encode ecological strategy signatures, distinguishing between primary degraders, trophic intermediates, and mucosal specialists. Integrating these insights, we propose a CBM-driven digestive pipeline linking substrate recognition and microbial attachment to primary hydrolysis, cross-feeding networks, and short-chain fatty acid production. This pipeline links CBM-mediated carbohydrate processing to host physiological outcomes, including gut barrier integrity, metabolic homeostasis, and excretion. Together, these findings highlight CBMs as important contributors to diet–microbe–host metabolic interactions and suggest that CBM profiles may help inform how dietary fibers are processed and fermented into downstream metabolites.

## Introduction

Dietary carbohydrates that escape host digestion enter the colon as structurally diverse fibers, including resistant starch (RS), arabinoxylans, β-glucans, pectins, and fructans. Their metabolic fate is largely influenced by the enzymatic machinery and binding modules encoded in the gut microbiome [1, 2]. While glycoside hydrolase (GH) and polysaccharide lyase (PL) families define catalytic capabilities, carbohydrate-binding modules (CBMs) can skew which substrates microbes can physically access, adhere to, and deconstruct under physiological conditions [3].

Genome-wide surveys of human gut isolates and metagenomes show that CBMs are abundant, diverse, and non-randomly distributed across taxa and niches. Analyses of representative microbiomes have revealed that carbohydrate-active enzyme (CAZymes), including CBM-tagged enzymes, are among the most enriched functional categories in the gut [4]. Tools such as dbCAN2 now allow systematic annotation of CBMs and their associated catalytic domains across thousands of genomes and metagenome-assembled genomes [5]. The data exposes an interesting pattern: some organisms encode large repertoires of starch-targeting CBMs, others specialize in plant cell-wall polysaccharides, and still others devote substantial capacity to host-derived mucin glycans.

Spatially resolved studies of the gastrointestinal tract have emphasized that microbial communities are organized along both longitudinal (oral cavity to distal colon) and radial (lumen to mucosal surface) axes, with strong nutrient and oxygen gradients [6, 7]. Mucin-associated communities, luminal fiber degraders, and crypt-resident populations occupy distinct physical and nutritional niches, implying that substrate-targeting domains such as CBMs are key determinants of where and how microbes persist.

Although early enzymology suggested CBMs were auxiliary, studies in physiologically relevant contexts now demonstrate that CBMs are essential for accessing insoluble and matrix-embedded glycans that dominate the gut fiber landscape [3, 8].

Here, we synthesize structural, genomic, and ecological evidence to examine how CBMs influence the routing of dietary carbohydrates through microbial networks and host metabolism. We describe cross-species CBM patterns along substrate and gastrointestinal spatial axes, draw on existing structural insights to highlight conserved binding features, and outline a CBM-driven digestive pipeline from ingestion to excretion. This highlights three recurring principles: substrate-axis specialization, compartment-specific CBM patterning along the gastrointestinal tract, and ecological strategy signatures associated with distinct CBM repertoires.

## Cross-Species CBM Patterns

### Global Architecture of CBMs across the Human Gut Microbiome

**Fig. 1:**
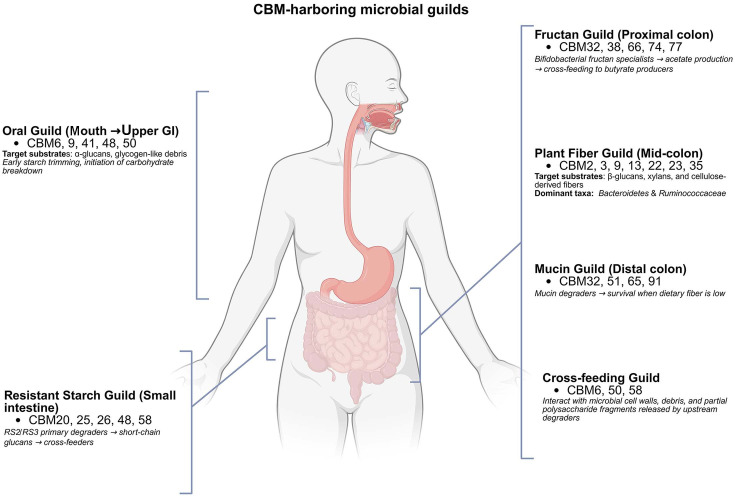
Spatial organization of carbohydrate-binding module (CBM) relevance along the human gastrointestinal tract. Schematic representation of the human gastrointestinal tract illustrating longitudinal compartments from the oral cavity to the distal colon. Major regions are annotated to indicate differences in substrate availability, transit time, and microbial density that influence carbohydrate utilization. The figure provides anatomical context for the compartment-specific distribution of dietary carbohydrates and host-derived glycans encountered by gut microorganisms, forming the spatial context within which CBMmediated substrate recognition and microbial foraging strategies operate. Created with BioRender.com.

CBMs display rich and non-random evolutionary patterning across the human gastrointestinal tract. This patterning reflects both the chemical complexity of dietary polysaccharides and the ecological stratification of the gut microbiota. Comparative analyses of gut genomes show that CBM families cluster into coherent groups corresponding to substrate specificity, gut compartment, and microbial foraging strategies [4, 5, 9]. These distributions are further shaped by the longitudinal organization of the gastrointestinal tract. Gradients in substrate availability, transit time, and microbial density create compartment-specific niches for CBM-mediated carbohydrate utilization (Fig. 1).

In *Bacteroidetes*, CBMs are often embedded within polysaccharide utilization loci (PULs) that encode coordinated systems for substrate binding, transport, and catalysis [10]. In contrast, *Firmicutes* commonly append CBMs to secreted amylases, pullulanases, and xylanases, forming multi-domain enzymes that couple substrate binding and catalysis [3, 8].

### Substrate-axis Specialization: CBMs Indicate Preferred Carbon Pools

Across gut and food-associated bacteria, distinct CBM portfolios align with preferred polysaccharide ‘axes’ and correlate with which carbon pools that individual strains are most likely to exploit. Resistant starch specialists such as *Ruminococcus bromii* devote much of their CAZyme arsenal to granular starch. These microorganisms encode CBM20/26-like starch-binding modules associated with GH13 amylases and pullulanases that attack intact starch granules. Metagenomic and fermentation studies consistently identify *R. bromii* as a keystone RS degrader in the human colon. Its presence stratifies community-level butyrogenic responses to Type II and III RS [8, 11-13]. More recent work extends this RS axis to selected *Bifidobacterium* species that encode CBM74 modules fused to GH13 amylases [14-16].

In contrast, fructan-focused bacteria preferentially invest in CBMs that recognize fructosyl residues of inulin and levan rather than glucose-rich starch surfaces. The founding CBM66 from the *Bacillus subtilis* β-fructosidase SacC binds terminal fructofuranosyl units on levan. This interaction selectively enhances levan hydrolysis by GH32 fructosidases without increasing activity on inulin [17]. Consistent with this specialization, CBM–GH32 architectures are enriched in fructan-utilizing taxa, positioning these organisms at the soluble-fructan end of the substrate axis [16].

A third, mucin-associated axis is defined by CBMs that target host glycoproteins rather than dietary polysaccharides. The *Clostridium perfringens* sialidase NanJ contains a CBM32 that binds terminal galactose or N-acetylgalactosamine and a CBM40 that recognizes sialic acid. Together, these modules form a coordinated system for extracting glycans from intestinal mucin [18, 19]. CBM32/CBM40 tandems are over-represented in mucin specialists and opportunistic pathogens that inhabit the mucus layer and often coincide with reduced capacity for plant cell wall glycan utilization [20, 21].

A comparable correspondence between CBM repertoires and substrate preference is observed regarding plant cell wall polysaccharides. In these systems, xylan- and β-glucan-binding CBMs are typically paired with GH10, GH43, GH5, or GH26 enzymes in *Bacteroides* and related taxa [22-25]. Together with starch-, fructan-, and mucin-associated modules, these plant cell wall CBMs form a multidimensional ‘CBM barcode’. This barcode is indicative of whether a strain behaves as a starch granule specialist, soluble fructan utilizer, mucin scavenger, or broad plant cell wall degrader *in vivo* [3].

### Compartmental Patterning across the Gastrointestinal Tract

Along the gastrointestinal tract, gradients in pH, bile acids, oxygen tension, transit time, and substrate availability create a series of partially segregated habitats rather than a single homogeneous lumen. Regional surveys in humans and animal models show systematic shifts in microbial density, composition, and metabolic output from the upper small intestine to the distal colon. Particularly sharp transitions occur at the ileocecal junction and along the proximal–distal colonic axis [26, 27]. Capsule-based profiling under physiological conditions further demonstrates coordinated changes in microbiota, proteins, and bile acids along this axis. These findings emphasize that glycan metabolism must be interpreted in a spatial context rather than inferred from stool alone [28].

The restructuring of the gut glycan landscape is mirrored by spatially restricted expansion of CAZyme repertoires. In the particulate phase of the proximal colon, RS granules act as discrete scaffolds that nucleate colonization by keystone degraders such as *R. bromii*. This species displays a superior capacity to colonize and depolymerize granular starch compared with other amylolytic bacteria [8]. Recent work on the *R. bromii* amylosome shows that multiple surface-associated enzymes are organized into constrained complexes tuned to RS microenvironments. These assemblies promote efficient local degradation and cross-feeding to surrounding fermenters [29]. The presence of starch-binding CBM families in these assemblies suggests that CBM-enriched amylosomes are retained at the interface between particulate and soluble carbohydrate pools, creating a spatially localized gateway for energy release from resistant glucans.

Closer to the epithelium, the architecture and glycosylation of the mucus layer define a second major carbohydrate niche. Colonic mucins form a stratified gel in which an inner, relatively sterile layer overlies an outer region enriched in complex O-glycans and colonized by specialized microbes [30]. Mucosa-associated communities are compositionally distinct from luminal consortia and are enriched in oxygen-tolerant taxa and mucin specialists such as *Akkermansia muciniphila*. In this species, binding to mucin depends on specific LacNAc-bearing O-glycan motifs that link colonization to fine-scale mucin glycan structure [26, 31, 32].

Structural studies of mucin-directed CBMs, particularly CBM32 modules appended to exo-N-acetylglucosaminidases and related enzymes, reveal multivalent recognition of terminal galactose and N-acetylglucosamine motifs characteristic of class III mucins. These findings support the idea that these CBMs partition enzymes to specific mucosal microdomains within the gut [18]. This compartmental patterning provides an essential spatial scaffold for interpreting CBM distributions in metagenomes and for constructing CBM-centered models of carbohydrate flow from ingestion to excretion.

### Ecological Strategy Signatures

Variation in CBM repertoires across gut microbes is non-random and aligns closely with ecological foraging strategies. As a result, CBMs can be read as molecular signatures of trophic roles. Primary degraders of recalcitrant substrates typically encode expansive and diverse CBM portfolios, including starch-, plant cell wall-, or fructan-directed families arranged in multidomain architectures on cell-surface enzymes and complexes. In RS ecosystems, *R. bromii* exemplifies this strategy as a keystone degrader that initiates particulate RS breakdown and enables growth of commensals unable to access granules directly. This role depends on dedicated surface complexes and high-affinity starch binding [8]. High-density CBM arrays promote adhesion to insoluble fibers, concentrate catalytic domains at substrate interfaces, and facilitate efficient deconstruction of otherwise inaccessible carbon pools. This is consistent with ecological models in which a minority of specialized species governs the release of fermentable oligosaccharides to the wider community [33].

In contrast, many secondary degraders and trophic opportunists occupy niches defined by soluble products released by these CBM-rich pioneers. They often possess few or no recognizable CBMs. Genomes of saccharolytic *Firmicutes* and *Bacteroidetes* that preferentially metabolize oligosaccharides or fermentation intermediates typically encode transporters and cytosolic glycoside hydrolases but sparse CBM content. This suggests reliance on public goods such as maltodextrins, fructo-oligosaccharides, and mucin fragments generated upstream in the trophic chain [34]. In this context, reduction or loss of CBM repertoires can be viewed as an adaptive trade-off favoring energetic efficiency and flexibility over direct access to insoluble substrates [33].

A third ecological signature characterizes mucosal specialists, which display narrow yet amplified sets of mucin-directed CBMs, particularly families such as CBM32 and CBM51 that recognize core mucin O-glycans. Comparative genomics and functional studies show that mucin-degrading taxa, including *A. muciniphila* and selected *Bacteroides* and *Ruminococcus* lineages, are enriched in CAZymes and CBMs targeting fucosylated, sialylated, and galactosylated mucin structures. This enrichment supports stable colonization of the mucus layer and foraging on host-derived glycans when dietary fiber is limited [35, 36]. These mucosal guilds are complemented by versatile generalists with hybrid CBM repertoires that combine plant fiber– and mucin-associated families. This architecture enables dynamic switching between dietary and host-derived substrates in response to environmental conditions [37]. These recurring associations between CBM composition, substrate preference, and trophic behavior are summarized in Table 1.

## Structural Biology of CBMs: A Continuous Architectural Spectrum and Shared Binding Logic

Early AlphaFold-based analyses established canonical CBM folds and binding modes, most notably the β-sandwich architectures of starch-binding CBM20s and the ligand-diverse CBM32s that recognize galactose, N-acetylgalactosamine, and N-acetylglucosamine epitopes [3, 38, 39]. AlphaFold2 further refines this view by revealing intra-family structural continua. Within CBM20, it predicts progressive expansion of starch-binding platforms through loop insertion, helical protrusions, and duplication of aromatic residues. These features correlate with functional differences between raw-starch-binding domains and modules preferring soluble maltooligosaccharides [3, 38, 39]. Structural variation is even more pronounced in CBM32, where a conserved β-sandwich core is paired with loop-level divergence that tunes specificity across galactose- and N-acetylhexosamine-containing ligands [18, 40-42].

More broadly, structural studies support a model in which CBMs populate a continuum of related β-rich architectures rather than discrete fold classes. This spectrum spans compact or truncated targeting motifs, classical single CBMs, and elaborate multidomain assemblies in which CBM-like units are integrated with catalytic and accessory domains [43-45]. Some regions previously annotated as CBMs lack stable carbohydrate-binding folds and instead function as flexible linkers or spacing elements. These observations show the importance of structural context and domain integration for distinguishing functional CBMs from auxiliary regions [44, 46, 47].

Because AlphaFold predictions are proteome-wide, a shared structural language of β-sandwich scaffolds, aromatic platforms, and polar complementarity can be traced across CBM families operating in distinct catalytic contexts. Comparisons of CBM20-like starch-binding domains with CBM48 modules from pullulanases and metabolic regulators reveal a continuous trajectory from globular, enzyme-tethered modules to elongated regulatory domains that embed carbohydrate recognition within broader protein-interaction surfaces [44, 48, 49]. Similarly, CBM6, CBM41, and CBM53 modules targeting mixed-linkage glucans and complex plant polysaccharides exhibit overlapping structural motifs. These similarities indicate that carbohydrate specificity often arises from subtle modification of shared structural templates rather than unrelated folds [3, 44, 50, 51]. This structural continuum provides the foundation for the subsequent sections, where CBM surface chemistries and domain arrangements are linked to ecological strategies, substrate preferences, and functional niches along the gastrointestinal tract.

### A Structural Continuum Rather than Discrete Families

The advent of AlphaFold2 and the AlphaFold Protein Structure Database enables large-scale, uniform comparisons of predicted CBM structures across genomes. These analyses reveal that many families populate a continuous structural landscape rather than isolated topological islands [50, 52]. When representative CBM20, CBM21, CBM32, CBM6, CBM9 and CBM44 domains are superposed in three dimensions, their architectures cluster around compact β-rich scaffolds. Differences arise primarily from loop elaboration, curvature of the binding surface and the positioning of aromatic residues, even when primary sequence identities fall below 15% [53]. Canonical structures of CBM20 and CBM32 already hinted at this shared template, showing distorted β-barrels or β-sandwich cores that cradle shallow clefts for starch or galactose recognition. More recent structures of CBM6 and CBM35 extend this theme to xylan and mannan binding modules [40, 54].

**Fig. 2:**
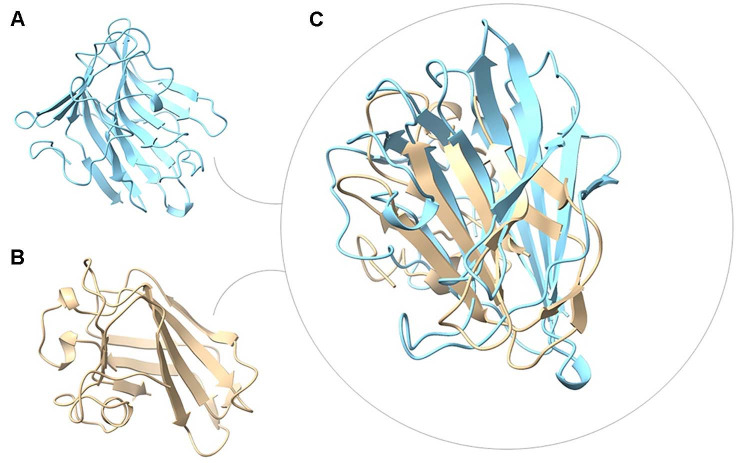
Structural comparison of mucin-associated carbohydrate-binding modules (CBMs) illustrates a continuum within a conserved scaffold. (**A**) Cartoon representation of CBM91 from *Roseburia intestinalis* (brown) and (**B**) CBM32 from *Bifidobacterium longum* (light blue) shown individually to highlight differences in strand length, loop architecture, and overall surface curvature. (**C**) Structural superposition of CBM91 and CBM32 aligned on the conserved β-sheet core reveals a shared β-sandwich scaffold, with structural divergence largely confined to surface-exposed loops. These variable regions are implicated in tuning glycan recognition and substrate specificity, supporting the view that mucin-associated CBMs occupy a structural continuum rather than discrete fold classes.

AlphaFold2 generalizes these observations beyond the limited set of crystallized CBMs by providing confident models for entire CBM repertoires across gut CAZymes. These models can be embedded into structure-based similarity networks [47, 50, 55]. Within these networks, families that appear unrelated at the sequence level occupy neighboring regions of structural space and can be connected by smooth trajectories of intermediate folds. This supports the view that CBMs form a topological continuum shaped by the physical demands of polysaccharide binding rather than by strict family boundaries. The continuum is characterized by recurring design solutions: β-sandwich or β-barrel cores, clusters of tryptophan or tyrosine that form aromatic stacking platforms, and surface grooves or pockets tuned in width and depth to accommodate linear glucans, branched mannans or kinked lacto-*N*-biose motifs [41, 56, 57]. From this perspective, CAZyme families remain valuable evolutionary labels, but the AlphaFold2 derived structural spectrum suggests that CBMs are better described as a mosaic of related architectures connected by incremental loop mutations and aromatic repositioning. The practical implication is that structural prediction and clustering can uncover latent binding relationships that are invisible to sequence-based annotation. This enables inference of CBM function across the entire gut microbiome using routine access to AlphaFold models [50, 52]. Structural superposition of representative CBM20, CBM32, CBM6, CBM44, CBM40 and CBM74 domains illustrates clustering around a shared β-rich scaffold with gradual variation in loop architecture and aromatic residue positioning. This supports the concept of a continuous structural spectrum rather than discrete family-specific folds. To illustrate shared architectural features across CBM families, representative CBM domains were selected based on canonical length, domain completeness and AlphaFold confidence. Domain-only AlphaFold models were superposed to highlight conserved scaffolds and aromatic binding features. Structural superposition of representative mucin-associated CBMs, including CBM32 and CBM91, reveals a conserved β-sandwich core with divergence largely confined to loop regions that tune ligand specificity, further supporting the continuum model (Fig. 2).

Key CBM reviews and databases already provide curated entry points for this continuum view. Early structural work was synthesized across multiple CBM families and highlighted how a limited set of stereochemical strategies is redeployed for diverse ligands [3]. Structure-guided approaches for predicting CBM specificity were later formalized, arguing that fold level features and aromatic topology can be more informative than raw sequence similarity [56]. Structural overlays of CBM32 with representative CBMs from distinct ecological guilds reveal a conserved β-sandwich core with functional divergence concentrated in surface-exposed regions (Fig. S1). Some CBM families, including LysM (CBM50), adopt loop-dominant architectures and lack a complete β-sandwich scaffold. Structural and solution studies demonstrate that bacterial LysM domains bind carbohydrates through additive, flexible surface interactions rather than stable higher-order assemblies. Notably, the presence of short β-elements and recurring strand–loop motifs in several LysM domains supports evolutionary plasticity and alternative binding solutions within the broader CBM structural continuum [58, 59].

### Conserved Aromatic Stacking Corridors: A Universal Binding Feature

Across diverse CBMs, the most conserved feature of the ligand-binding surface is not a specific sequence motif but a shared architectural principle: extended corridors of aromatic side chains. These corridors create low-energy landscapes for polysaccharide rings to dock, slide, and reorient. Early structural studies showed that many CBM families employ planar or grooved binding faces enriched in tryptophan, tyrosine, or phenylalanine residues, enabling carbohydrate recognition through CH–π stacking and hydrophobic contacts despite divergent folds and biological contexts [3]. These aromatic stacking corridors align with the periodicity of pyranose rings and provide a general solution for binding chemically similar yet structurally heterogeneous glycans. Specificity is fine-tuned via flanking hydrogen bonds and electrostatic interactions.

Biophysical and computational analyses have demonstrated that CH–π interactions contribute binding energies comparable to hydrogen bonds and are geometrically permissive. This explains why CBM binding sites often comprise multiple aromatic residues arranged in series, supporting ligand sliding and positional flexibility [60-63]. Archetypal examples include type A CBMs targeting crystalline polysaccharides and starch-binding CBM20 and CBM21 modules. These domains present extended aromatic surfaces that accommodate helical or linear glucan chains while permitting limited substrate mobility [64]. In CBMs that bind internal glycosidic linkages, such as families targeting xylans or mixed-linkage glucans, aromatic corridors are embedded within shallow grooves rather than flat faces, but rely on the same stacking logic [65-67].

**Fig. 3:**
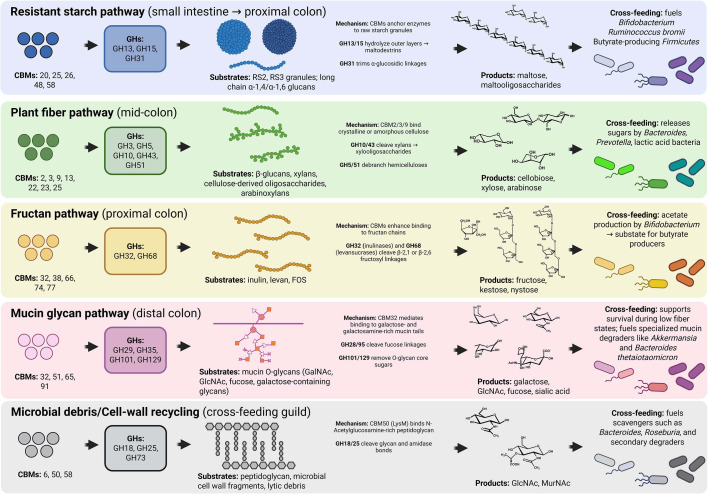
CBM-guided substrate engagement strategies. Schematic illustration of how conserved carbohydrate-binding module (CBM) scaffolds are deployed as distinct substrate engagement strategies across carbohydrate contexts. (**A**) Flat-faced CBMs associated with resistant starch and plant cell wall polysaccharides mediate surface anchoring to insoluble substrates, increasing local enzyme concentration and promoting efficient hydrolysis. (**B**) Pocket-forming CBMs involved in fructan metabolism facilitate capture of flexible soluble polymers, enhancing substrate encounter frequency in the lumen. (**C**) Loop-enriched CBMs associated with mucin glycan degradation enable selective recognition of decorated host-derived epitopes. Together, these strategies demonstrate how variation on a conserved β-sandwich scaffold tunes functional engagement without invoking discrete binding modes. Created with BioRender.com.

Mucin-associated CBM32s illustrate how this principle is repurposed for selective recognition of terminal epitopes. These modules use compact aromatic platforms combined with polar residues to bind galactose- and N-acetylhexosamine-containing glycans, with loop rearrangements modulating specificity across related ligands [18, 68, 69]. Collectively, these observations support a unifying model in which aromatic stacking corridors constitute a universal CBM binding strategy, adaptable to surface anchoring, pocket-based capture, or epitope recognition depending on substrate chemistry and ecological context (Fig. 3).

### Substrate Sliding Channels and Processivity Elements

Processive glycosidases achieve high catalytic efficiency by threading a single polysaccharide chain through extended binding tunnels, enabling multiple catalytic steps before dissociation. Studies of archetypal cellobiohydrolases established this model, in which conserved aromatic residues line substrate-binding tunnels to stabilize successive sugar units during chain advancement [70, 71]. Single-molecule and simulation analyses further indicate that dissociation occurs via stepwise dethreading rather than simple unbinding, highlighting the physical reality of sliding pathways rather than point-binding pockets [70-74].

An analogous principle operates in α-glucan hydrolases and transferases acting on starch-like substrates. Structural and mutational studies show that many α-amylases, branching enzymes, and debranching enzymes possess secondary glucan-contacting surfaces that extend from the catalytic cleft. These features form shallow grooves or ridges along which glucan chains can align [75, 76]. These tracks may be encoded by discrete CBMs (*e.g.*, CBM20, CBM41, CBM48, CBM68) or by surface-binding sites embedded within catalytic domains, but in both cases they support substrate sliding rather than static adsorption [77].

Molecular simulations and mutational analyses support this sliding model. For example, CBM20 contains multiple aromatic-centered binding sites that can bind and locally destabilize amylose helices while remaining attached. This allows the glucan chain to reposition along the CBM surface as the structure unwinds and is delivered to the catalytic site [78]. Consistent with this view, disruption of key aromatic or polar residues in CBM68 domains reduces both oligosaccharide binding and catalytic efficiency in pullulanases, directly linking CBM integrity to α-glucan processivity [79].

At the level of full-length enzymes, domain-architecture studies reinforce the coupling between CBMs and substrate-binding tunnels. Alteration of CBM-rich regions in pullulanases and related GH13 and GH57 enzymes shifts product-length distributions and catalytic behavior. This indicates that distal binding modules influence how far a chain can slide and when it is released, not merely how strongly the enzyme adheres to the substrate [75, 76, 80, 81].

### A unified model for CBM ligand recognition

Across CBM families and proto-CBM candidates, AlphaFold2 and experimental structures converge on a common design logic for carbohydrate recognition. First, aromatic stacking networks formed by tryptophan, tyrosine, or phenylalanine residues create planar or gently curved surfaces that support CH–π interactions with sugar rings. This principle is exemplified by the dual binding sites of CBM20 in glucoamylase [82]. Second, flexible loop regions tune specificity and affinity by altering cleft depth, contour, and hydrogen-bonding potential. This mechanism is highlighted in comparative analyses of glycan binding by *Bacteroidetes* Sus-like systems [83]. Third, continuous trenches or shallow cavities running along the protein surface provide potential sliding tracks for polysaccharide chains. These features suggest that CBMs do not simply pin substrates in place but guide them across catalytic or multi-enzyme assemblies. This recurrent triad of aromatic stacking, loop plasticity, and chain-guiding topology supports a unified model in which CBMs are variations on a shared structural solution to the challenge of binding extended, chemically heterogeneous polysaccharides [56].

### Implications for Fiber Utilization, Microbial Ecology and Enzyme Engineering

Recognizing CBMs and proto-CBMs as points along a structural continuum has practical consequences for understanding and manipulating gut carbohydrate metabolism. Primary degraders of resistant starch, such as *R. bromii*, combine starch-directed CBMs with cell-surface localization to colonize and erode granules [8]. Mucin specialists use CBM32 and CBM51 modules, together with specialized mucinases, to harvest host O-glycans [35, 84]. For enzyme engineering, domain swapping experiments in Sus-like and pullulanase systems show that altering CBM composition can shift both substrate range and product-length distributions [83]. AlphaFold2 enables *in silico* discovery of uncharacterized CBMs and proto-CBMs with novel geometries. Together, these advances provide a structural basis for designing CAZymes, probiotics, and fiber formulations that are tuned to specific CBM repertoires and ecological niches [52]. The major structural strategies by which CBMs engage carbohydrates across digestive contexts are summarized in Table 2.

## The CBM-Driven Digestive Pipeline: Linking Substrate Recognition to Microbial Ecosystems and Host Metabolism

**Fig. 4:**
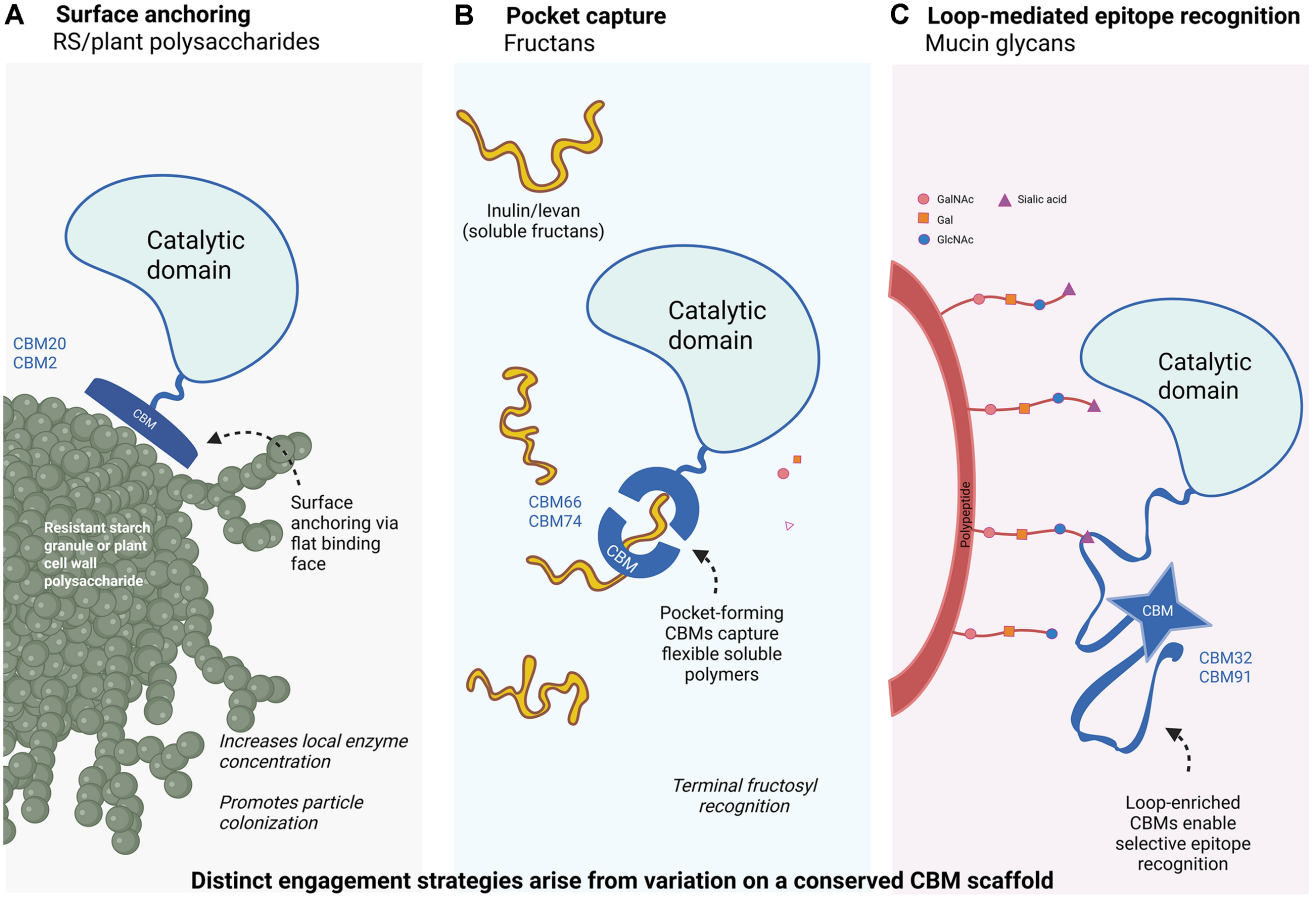
CBM-driven digestive pipeline for dietary and host-derived carbohydrates in the human gut. Overview of major carbohydrate processing pathways organized by substrate class and gut location, including resistant starch, plant cell wall polysaccharides, fructans, mucin O-glycans, and microbial cell-wall debris. For each pathway, representative CBM families, associated glycoside hydrolase (GH) families, primary substrates, enzymatic mechanisms, and resulting oligosaccharide or monosaccharide products are indicated. The schematic also summarizes downstream cross-feeding interactions among microbial groups associated with each pathway along the gastrointestinal tract. Created with BioRender.com.

Dietary carbohydrates traverse the gastrointestinal tract through a sequence of stages shaped by polymer chemistry, enzyme repertoires, and the CBMs that contribute to substrate access. To integrate molecular binding specificity, microbial ecology, and metabolic outcomes, we outline a CBM-driven digestive pipeline that traces the fate of major dietary and host-derived carbohydrates along the gastrointestinal tract (Fig. 4). This links CBM family composition to substrate access, enzymatic processing, cross-feeding networks, and downstream metabolite production. In this model, CBMs operate as access-bias modules that prioritize substrate engagement in specific microorganisms. Through this bias, CBMs influence how trophic networks assemble and which metabolites ultimately interact with host tissues. The CBM-driven digestive pipeline integrates molecular binding events, ecological dynamics, and host physiology, complementing existing models of glycan utilization [83, 85]. For clarity, the sequential stages of CBM-mediated carbohydrate processing along the gastrointestinal tract are summarized in Table 3. This pipeline is intended as an integrative and hypothesis-generating model that synthesizes existing molecular, ecological, and metabolic evidence, rather than a direct causal model linking individual CBMs to host physiological outcomes.

### Stage One: Ingestion and Initial CBM Filtered Access

When food enters the gut, the structural heterogeneity of polysaccharides interacts with the resident CBM landscape. Resistant starch granules are preferentially targeted by CBM20 and related families, which confer high affinity for granular starch and support colonization by organisms such as *R. bromii* [8]. Fructans, including inulin and shorter fructooligosaccharides, are primarily handled by GH32 enzymes often equipped with fructan directed CBMs in *Bifidobacterium* species. This is consistent with fructan focused bifidogenic effects summarized in clinical and mechanistic reviews of complex glycan catabolism [83]. Plant cell wall polysaccharides engage CBM families that recognize xylan, arabinoxylan, and mixed-linkage glucans within *Bacteroides* and *Prevotella* PULs [83]. Host mucin glycans are bound by CBM32 and related modules in mucin-degrading taxa such as *A. muciniphila* [84]. At this earliest stage, CBM repertoires effectively partition dietary and host derived substrates among microbial guilds.

### Stage Two: Early Digestion and Exposure of New Binding Surfaces

In the oral cavity and upper gastrointestinal tract, salivary and pancreatic enzymes initiate partial depolymerization of complex carbohydrates. These enzymes trim α-1,4 and α-1,6 linkages and disrupt crystalline packing in starch and some plant glycans. As a result, hydration increases, new chain ends are generated, and internal polysaccharide segments become exposed. These newly accessible regions later interact with CBMs in the distal gut. Structural studies of human and microbial amylases indicate that flexible loops near the active site can engage substrate beyond the catalytic pocket [83, 86, 87]. AlphaFold2 extends this concept by predicting CBM-like loops and microdomains in several oral GH families. As such, even in regions where annotated CBMs are rare, latent binding motifs may influence how substrates are pre-processed and presented downstream.

### Stage Three: Primary Degradation in the Proximal Colon

The proximal colon hosts primary degraders that rely heavily on CBMs to colonize and deconstruct complex, often insoluble substrates. *Ruminococcus bromii* attaches to and bores into resistant starch particles, a phenotype linked to its exceptional starch degradation capacity in human colonic ecosystems [8, 29, 88]. *Bacteroides* species use Sus-like PULs that combine surface binding proteins and CBM equipped glycosidases to harvest a wide range of dietary and host glycans [89-91]. In these systems, CBMs increase local substrate concentration at the cell surface and align glycan chains for efficient hydrolysis, while multi-CBM architectures further enhance adhesion to particulate fibers. The products of this stage are soluble oligosaccharides and smaller polymers that distribute into the broader community.

### Stage Four: Cross-Feeding Networks Shaped by CBM Mediated Release

Secondary degraders, many of which lack CBMs altogether, depend on the soluble oligosaccharides liberated by primary degraders. Controlled human and animal studies of fructan supplementation demonstrate substantial bifidogenic responses. These outcomes are consistent with the idea that CBM enabled GH32 activity in primary degraders produces fructan fragments that feed Bifidobacteria [92-94]. When dietary fiber is scarce, mucin degrading bacteria such as *A. muciniphila* expand and upregulate mucin targeting CAZymes, eroding the mucus barrier [84, 95]. These shifts illustrate how CBM-guided access to dietary glycans or host mucus can shape cross feeding, niche succession, and competition among mucus associated and lumen adapted populations.

### Stage Five: Fermentation and Short-Chain Fatty Acid Production

The substrate pool generated by CBM-dependent degradation strongly influences which fermentation pathways dominate in the gut. As a result, CBM activity indirectly shapes the profiles of short-chain fatty acids (SCFAs) produced. Acetate, propionate, and butyrate arise from overlapping but distinct microbial guilds [96, 97]. Butyrate producing taxa such as *Faecalibacterium prausnitzii* have been shown to exert pronounced anti-inflammatory and barrier-supporting effects [98, 99]. SCFAs modulate epithelial tight junctions, energy metabolism, immune signaling, and neuroimmune pathways [97, 100]. Since CBMs influence which fibers are initially accessible and how they are fragmented, they can indirectly shape SCFA profiles through effects on microbial trophic structure and substrate flow. Extensive literature links these SCFAs to downstream host effects.

### Stage Six: Excretion as a Readout of CBM Pathway Performance

The composition of fecal material provides an integrated readout of CBM mediated digestion across the length of the gut. Persistent resistant starch in stool is consistent with limited engagement by CBM20-rich starch degraders such as *R. bromii*. In contrast, accumulation of mucin fragments and host-derived glycans is consistent with increased reliance on CBM32 and related mucin-foraging systems. These patterns are consistent with mucus degradation and barrier erosion under fiber-deprived conditions [95]. Emerging glycomic and metagenomic approaches now profile fecal oligosaccharides and annotate the corresponding CAZyme and CBM repertoires. Such methods link excreted structures to upstream glycan degradation potential as in diet-linked glycan degradation analyses such as GlyDeR [83, 101]. In this view, stool is not simply waste but an ecological and biochemical fingerprint of CBM-directed carbohydrate processing.

**Fig. 5:**
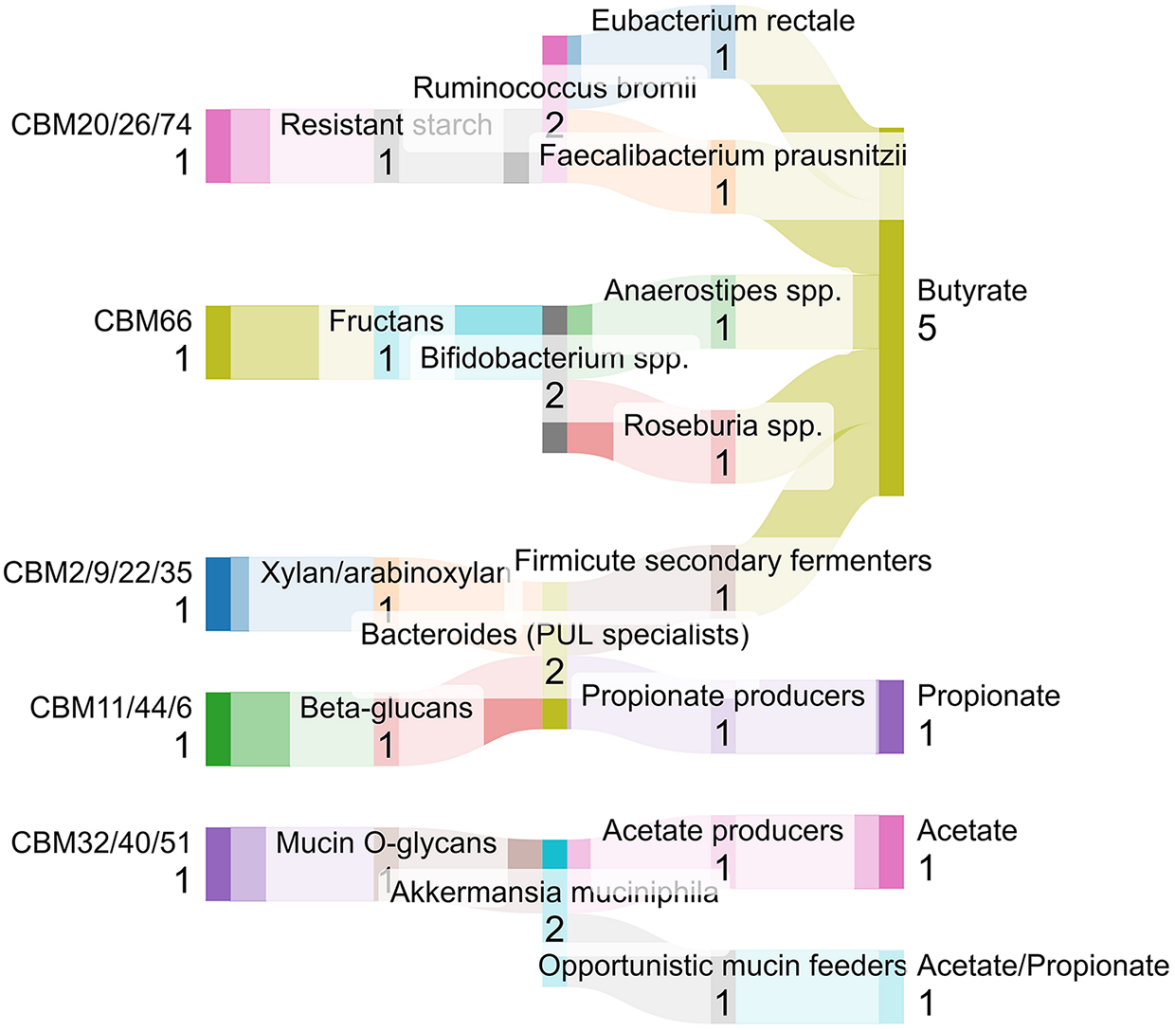
CBM-guided trophic routing of dietary and host-derived carbohydrates in the human gut. Conceptual schematic illustrating how CBM repertoires bias substrate engagement and microbial succession along the gastrointestinal tract. Distinct CBM families preferentially associate enzymes with resistant starch, fructans, plant cell-wall polysaccharides, or host mucin O-glycans, shaping which microbial guilds are most likely to initiate degradation under physiological conditions. Soluble products released by these primary degraders support secondary fermenters that often lack CBMs, contributing to characteristic short-chain fatty acid (SCFA) profiles, including acetate, propionate, and butyrate. All flows are schematic and qualitative, shown with equal weight to illustrate conceptual routing rather than quantitative flux, relative abundance, or measured metabolic rates.

Altogether, these stages define a CBM-driven digestive pipeline in which CBMs act as central organizers of the diet-microbe-host axis. At the molecular level, CBMs encode binding specificities and geometries that initiate substrate recognition, as exemplified by high resolution CBM20 structures [52, 82]. At the ecological level, CBM repertoires partition substrates among primary degraders, cross feeders, and mucin specialists [35, 83]. At the metabolic level, CBM-guided routing of fibers into specific trophic chains can shape SCFA profiles and glycan intermediates. These metabolites are known to influence barrier integrity, systemic inflammation, and gut–brain communication [96, 100]. This basis motivates a shift away from defining dietary fibers solely by monosaccharide composition or solubility. Instead, dietary carbohydrates are defined by the CBM repertoires capable of recognizing them and by the ecological and metabolic cascades that follow. These CBM-mediated stages can be integrated into a unified trophic routing context in which CBMs bias substrate encounter probabilities and spatial enzyme localization, thereby shaping, but not strictly determining, microbial succession and downstream metabolic outcomes (Fig. 5).

## Beyond Carbohydrates: The Modular Nutrient-Binding Universe

CBMs are the best characterized nutrient targeting domains in the human gut microbiome, but they are only one part of a much broader modular nutrient binding universe. Across bacteria, archaea, and eukaryotes, evolution has repeatedly converged on a shared strategy of modularity, in which discrete non-catalytic domains are appended to catalytic cores to improve substrate localization, specificity, and metabolic efficiency. Alongside CBMs, organisms deploy specialized modules that bind proteins, lipids, vitamins, cofactors, and polyphenols, producing a higher order nutrient binding super network that steers where energy and micronutrients flow in complex diets. CBMs exemplify a broader evolutionary strategy in which modular non-catalytic domains favor nutrient access. Similar principles operate across protein, lipid, and micronutrient metabolism, underscoring that substrate binding, not catalysis alone, often determines ecological success [102, 103].

## Future Predictions and Conclusion

Carbohydrate-binding modules are not peripheral enzymatic appendages but key molecular determinants that influence how dietary carbohydrates are accessed and processed by gut microbes. By mapping CBMs to trophic networks, the manuscript proposes testable predictive rules linking CBM-guided degradation to acetate, propionate, and butyrate formation. These insights motivate future exploration of CBM-guided strategies aimed at modulating microbial fiber utilization in contexts such as metabolic syndrome, inflammatory disease, mucin-barrier dysfunction, and neuroimmune imbalance.

From an engineering perspective, CBM-guided design may offer routes to regulate fiber breakdown, enabling engineered probiotics or synthetic CAZyme systems to target defined glycan structures and modulate downstream SCFA profiles. Within this CBM-driven digestive pipeline, excreted fiber fragments and mucin residues may serve as candidate biomarkers of upstream CBM-mediated processing. Collectively, these insights position CBMs as central organizers of the diet–microbiome–host interface and establish a roadmap for future work in precision nutrition, microbiome engineering, metabolic modelling, and evolutionary ecology.

## Supplemental Materials

Supplementary data for this paper are available on-line only at http://jmb.or.kr.



## Figures and Tables

**Table 1:** CBM families as indicators of substrate specialization and ecological role in the human gut.

Dominant CBM family (examples)	Primary carbohydrate target	Typical associated GH families	Representative gut taxa	Predicted ecological role
CBM20, CBM26, CBM74	Resistant starch (granular, semi-crystalline)	GH13, GH57, GH77	*Ruminococcus bromii*, *Bifidobacterium adolescentis*	Keystone primary degrader; initiates particulate starch breakdown
CBM2, CBM9, CBM15, CBM22, CBM35	Plant cell wall polysaccharides (xylan, arabinoxylan, β-glucans)	GH10, GH43, GH5, GH26	*Bacteroides ovatus*, *Bac. thetaiotaomicron*	Broad-spectrum plant fiber degrader; PUL-driven generalist
CBM6, CBM41, CBM44	Mixed-linkage glucans and recycled microbial polysaccharides	GH16, GH3, GH5	*Bacteroides fragilis*, *Bac. uniformis*	Secondary degrader; recycling of soluble glucans
CBM66, CBM32 (fructan-adjacent variants)	Fructans (inulin, levan, FOS)	GH32, GH68	*Bifidobacterium longum*, *Anaerostipes hadrus*	Soluble fructan specialist; cross-feeding hub
CBM32, CBM40, CBM51	Host mucin O-glycans	GH89, GH95, GH33	*Akkermansia muciniphila*, *Clostridium perfringens*	Mucosal specialist; host-glycan forager
CBM-poor or CBM-absent	Soluble oligosaccharides only	Cytosolic GHs, transporters	*Faecalibacterium prausnitzii*	Secondary fermenter; SCFA producer

FOS, fructooligosaccharide; GH, glycoside hydrolase; PUL, polysaccharide utilization locus; SCFA, short-chain fatty acids.

**Table 2:** Structural strategies used by CBMs to engage carbohydrates across digestive contexts.

CBM structural feature	Binding geometry	Typical substrates	Functional consequence	Representative CBM families
Flat aromatic face	Planar surface anchoring	Crystalline cellulose, resistant starch granules	Stable attachment to insoluble particles; increased local enzyme concentration	CBM20, CBM2, CBM1
Shallow groove / trench	Linear or gently curved channel	Soluble glucans, β-glucans, xylans	Substrate sliding; enhanced processivity	CBM6, CBM44, CBM41
Pocket-forming loops	Enclosed or semi-enclosed pocket	Fructans, terminal fructosyl units	Capture of flexible polymers; high affinity for soluble substrates	CBM66, CBM32 (fructan-active variants)
Loop-enriched recognition surface	Decorated, adaptable binding site	Mucin O-glycans, host glycoconjugates	Epitope-specific host glycan recognition	CBM32, CBM40
Multi-CBM arrays	Repeated binding modules	Particulate polysaccharide matrices	Avidity effects; spatial retention at substrate interface	CBM20 tandems, CBM74 clusters
Proto-CBM microdomains	Partial β-sandwich or aromatic patches	Diverse, low-affinity glycans	Latent binding; evolutionary precursors to full CBMs	AlphaFold-predicted CBM-like loops

**Table 3:** Sequential stages of CBM-mediated carbohydrate processing in the human gastrointestinal tract.

Stage	Biological process	Role of CBMs
1	Ingestion of dietary fibers	Substrate-specific CBM recognition (RS, fructans, plant cell wall, mucin)
2	Early digestion (oral → small intestine)	Exposure of new binding surfaces; CBM-like microdomains increase accessibility
3	Primary degradation (proximal colon)	CBMs anchor degraders to insoluble fibers; enable enzymatic hydrolysis
4	Cross-feeding interactions	CBM-driven release of soluble oligos fuels secondary degraders lacking CBMs
5	Fermentation and SCFA formation	CBM-guided substrate access can bias which fragments enter acetate/propionate/butyrate pathways
6	Excretion	Undigested structures can reflect incomplete engagement by CBM-associated degradation pathways (*e.g.*, RS granules, mucin fragments, oligosaccharides)
7	Host physiology	SCFA &amp; glycan intermediates produced downstream may provide mechanistic links to host outcomes (barrier function, immune signaling, metabolism)

RS, resistant starch; SCFA, short-chain fatty acids.

## References

[1] Flint HJ, Scott KP, Louis P, Duncan SH (2012). The role of the gut microbiota in nutrition and health. Nat. Rev. Gastroenterol. Hepatol..

[2] Makki K, Deehan EC, Walter J, Bäckhed F (2018). The impact of dietary fiber on gut microbiota in host health and disease. Cell Host Microbe.

[3] Boraston AB, Bolam DN, Gilbert HJ, Davies GJ (2004). Carbohydrate-binding modules: fine-tuning polysaccharide recognition. Biochem. J..

[4] Kaoutari AE, Armougom F, Gordon JI, Raoult D, Henrissat B (2013). The abundance and variety of carbohydrate-active enzymes in the human gut microbiota. Nat. Rev. Microbiol..

[5] Zhang H, Yohe T, Huang L, Entwistle S, Wu P, Yang Z (2018). dbCAN2: a meta server for automated carbohydrate-active enzyme annotation. Nucleic Acids Res..

[6] Donaldson GP, Lee SM, Mazmanian SK (2016). Gut biogeography of the bacterial microbiota. Nat. Rev. Microbiol..

[7] Tropini C, Earle KA, Huang KC, Sonnenburg JL (2017). The gut microbiome: connecting spatial organization to function. Cell Host Microbe.

[8] Ze X, Duncan SH, Louis P, Flint HJ (2012). *Ruminococcus bromii* is a keystone species for the degradation of resistant starch in the human colon. ISME J..

[9] Fujimoto Z (2013). Structure and function of carbohydrate-binding module families 13 and 42 of glycoside hydrolases, comprising a β-trefoil fold. Biosci. Biotechnol. Biochem...

[10] Martens EC, Lowe EC, Chiang H, Pudlo NA, Wu M, McNulty NP (2011). Recognition and degradation of plant cell wall polysaccharides by two human gut symbionts. PLoS Biol..

[11] Mukhopadhya I, Moraïs S, Laverde‐Gomez J, Sheridan PO, Walker AW, Kelly W (2018). Sporulation capability and amylosome conservation among diverse human colonic and rumen isolates of the keystone starch‐degrader *Ruminococcus bromii*. Environ. Microbiol..

[12] Vital M, Howe A, Bergeron N, Krauss RM, Jansson JK, Tiedje JM (2018). Metagenomic insights into the degradation of resistant starch by human gut microbiota. Appl. Environ. Microbiol..

[13] Bendiks ZA, Knudsen KE, Keenan MJ, Marco ML (2020). Conserved and variable responses of the gut microbiome to resistant starch type 2. Nutr. Res..

[14] Dobranowski PA, Stintzi A (2021). Resistant starch, microbiome, and precision modulation. Gut Microbes.

[15] Valk V, Lammerts van Bueren A, van der Kaaij RM, Dijkhuizen L (2016). Carbohydrate‐binding module 74 is a novel starch‐binding domain associated with large and multidomain α‐amylase enzymes. FEBS J..

[16] Jung DH, Park CS (2023). Resistant starch utilization by *Bifidobacterium*, the beneficial human gut bacteria. Food Sci. Biotechnol..

[17] Cuskin F, Flint JE, Gloster TM, Morland C, Basl A, Henrissat B (2012). How nature can exploit nonspecific catalytic and carbohydrate binding modules to create enzymatic specificity. Proc. Natl. Acad. Sci. USA.

[18] Ficko-Blean E, Stuart CP, Suits MD, Cid M, Tessier M, Woods RJ (2012). Carbohydrate recognition by an architecturally complex α-N-acetylglucosaminidase from *Clostridium perfringens*. PLoS One.

[19] Boraston AB, Ficko-Blean E, Healey M (2007). Carbohydrate recognition by a large sialidase toxin from *Clostridium perfringens*. Biochemistry.

[20] Connaris H, Crocker PR, Taylor GL (2009). Enhancing the receptor affinity of the sialic acid-binding domain of *Vibrio cholerae* sialidase through multivalency. J. Biol. Chem..

[21] Ribeiro JP, Pau W, Pifferi C, Renaudet O, Varrot A, Mahal LK (2016). Characterization of a high-affinity sialic acid-specific CBM40 from *Clostridium perfringens* and engineering of a divalent form. Biochem. J..

[22] McCartney L, Blake AW, Flint J, Bolam DN, Boraston AB, Gilbert HJ (2006). Differential recognition of plant cell walls by microbial xylan-specific carbohydrate-binding modules. Proc. Natl. Acad. Sci. USA.

[23] Szab L, Jamal S, Xie H, Charnock SJ, Bolam DN, Gilbert HJ (2001). Structure of a family 15 carbohydrate-binding module in complex with xylopentaose: evidence that xylan binds in an approximate 3-fold helical conformation. J. Biol. Chem..

[24] Carvalho AL, Goyal A, Prates JA, Bolam DN, Gilbert HJ, Pires VM (2004). The family 11 carbohydrate-binding module of *Clostridium thermocellum* Lic26A-Cel5E accommodates β-1, 4-and β-1, 3-1, 4-mixed linked glucans at a single binding site. J. Biol. Chem..

[25] Najmudin S, Guerreiro CI, Carvalho AL, Prates JA, Correia MA, Alves VD (2006). Xyloglucan is recognized by carbohydrate-binding modules that interact with β-glucan chains. J. Biol. Chem..

[26] Yasuda K, Oh K, Ren B, Tickle TL, Franzosa EA, Wachtman LM (2015). Biogeography of the intestinal mucosal and lumenal microbiome in the rhesus macaque. Cell Host Microbe.

[27] Martinez-Guryn K, Leone V, Chang EB (2019). Regional diversity of the gastrointestinal microbiome. Cell Host Microbe.

[28] Shalon D, Culver RN, Grembi JA, Folz J, Treit PV, Shi H (2023). Profiling the human intestinal environment under physiological conditions. Nature.

[29] Wimmer BH, Moraïs S, Amit I, Tovar-Herrera O, Tatli M, Trautwein-Schult A (2025). Spatial constraints drive amylosome-mediated resistant starch degradation by *Ruminococcus bromii* in the human colon. Nat Commun..

[30] Luis AS, Hansson GC (2023). Intestinal mucus and their glycans: A habitat for thriving microbiota. Cell Host Microbe..

[31] Wu M, Li P, Li J, An Y, Wang M, Zhong G (2019). The differences between luminal microbiota and mucosal microbiota in mice. J. Microbiol. Biotechnol..

[32] Elzinga J, Narimatsu Y, de Haan N, Clausen H, de Vos WM, Tytgat HL (2024). Binding of *Akkermansia muciniphila* to mucin is O-glycan specific. Nat. Commun..

[33] Flint HJ, Scott KP, Duncan SH, Louis P, Forano E (2012). Microbial degradation of complex carbohydrates in the gut. Gut Microbes.

[34] Morris JJ, Lenski RE, Zinser ER. 2012. The Black Queen Hypothesis. *mBio* **3.** https://doi.org/10.1128/mbio.00036-12. 10.1128/mBio.00036-12 22448042 PMC3315703

[35] Tailford LE, Crost EH, Kavanaugh D, Juge N (2015). Mucin glycan foraging in the human gut microbiome. Front. Genet..

[36] Glover JS, Ticer TD, Engevik MA (2022). Characterizing the mucin-degrading capacity of the human gut microbiota. Scie. Rep..

[37] Ndeh D, Gilbert HJ (2018). Biochemistry of complex glycan depolymerisation by the human gut microbiota. FEMS Microbiol. Rev..

[38] Christiansen C, Abou Hachem M, Janeček Š, Viksø‐Nielsen A, Blennow A, Svensson B (2009). The carbohydrate‐binding module family 20-diversity, structure, and function. FEBS J..

[39] Shoseyov O, Shani Z, Levy I (2006). Carbohydrate binding modules: biochemical properties and novel applications. Microbiol. Mol. Biol. Rev..

[40] Ficko-Blean E, Boraston AB (2009). N-acetylglucosamine recognition by a family 32 carbohydrate-binding module from *Clostridium perfringens* NagH. J. Mol. Biol..

[41] Zhang X, Sunagawa N, Kashima T, Igarashi K, Miyanaga A, Fushinobu S. 2025. Structural insights into lacto‐N‐biose I recognition by a family 32 carbohydrate‐binding module from *Bifidobacterium bifidum*. *FEBS Lett.* https://doi.org/10.1002/1873-3468.70217 10.1002/1873-3468.70217 41204437 PMC12926856

[42] Grondin JM, Duan D, Kirlin AC, Abe KT, Chitayat S, Spencer HL (2017). Diverse modes of galacto-specific carbohydrate recognition by a family 31 glycoside hydrolase from *Clostridium perfringens*. PLoS One.

[43] Duan CJ, Feng YL, Cao QL, Huang MY, Feng JX (2016). Identification of a novel family of carbohydrate-binding modules with broad ligand specificity. Scientific Reports..

[44] Sidar A, Albuquerque ED, Voshol GP, Ram AF, Vijgenboom E, Punt PJ (2020). Carbohydrate binding modules: diversity of domain architecture in amylases and cellulases from filamentous microorganisms. Front. Bioeng. Biotechnol..

[45] Elfmann C, Stülke J (2023). PAE viewer: a webserver for the interactive visualization of the predicted aligned error for multimer structure predictions and crosslinks. Nucleic Acids Res..

[46] Gao M, Coletti M, Davidson RB, Prout R, Abraham S, Hernández B, *et al*. 2022. Presented at the 2022 IEEE International Parallel and Distributed Processing Symposium Workshops (IPDPSW). https://doi.org/10.1109/IPDPSW55747.2022.00045. 10.1109/IPDPSW55747.2022.00045

[47] Bertoline LM, Lima AN, Krieger JE, Teixeira SK (2023). Before and after AlphaFold2: an overview of protein structure prediction. Front. Bioinformatics.

[48] Machovič M, Janeček Š (2008). Domain evolution in the GH13 pullulanase subfamily with focus on the carbohydrate-binding module family 48. Biologia (Lahore, Pakistan).

[49] Cifuente JO, Colleoni C, Kalscheuer R, Guerin ME (2024). Architecture, function, regulation, and evolution of α-glucans metabolic enzymes in prokaryotes. Chem. Rev..

[50] Varadi M, Anyango S, Deshpande M, Nair S, Natassia C, Yordanova G (2021). AlphaFold Protein Structure Database: massively expanding the structural coverage of protein-sequence space with high-accuracy models. Nucleic Acids Res..

[51] Abramson J, Adler J, Dunger J, Evans R, Green T, Pritzel A (2024). Accurate structure prediction of biomolecular interactions with AlphaFold 3. Nature..

[52] Jumper J, Evans R, Pritzel A, Green T, Figurnov M, Ronneberger O (2021). Highly accurate protein structure prediction with AlphaFold. Nature.

[53] Pires VM, Pereira PM, Brás JL, Correia M, Cardoso V, Bule P (2017). Stability and ligand promiscuity of type A carbohydrate-binding modules are illustrated by the structure of *Spirochaeta thermophila* StCBM64C. J. Biol. Chem..

[54] Mizutani K, Fernandes VO, Karita S, Luís AS, Sakka M, Kimura T (2012). Influence of a mannan binding family 32 carbohydrate binding module on the activity of the appended mannanase. Appl. Environ. Microbiol..

[55] Varadi M, Velankar S (2023). The impact of alphafold protein structure database on the fields of life sciences. Proteomics..

[56] Abbott DW, van Bueren AL (2014). Using structure to inform carbohydrate binding module function. Curr. Opin. Struct. Biol..

[57] Abbott DW, Eirín-López JM, Boraston AB (2008). Insight into ligand diversity and novel biological roles for family 32 carbohydrate-binding modules. Mol. Biol. Evol..

[58] Mesnage S, Dellarole M, Baxter NJ, Rouget JB, Dimitrov JD, Wang N (2014). Molecular basis for bacterial peptidoglycan recognition by LysM domains. Nat. Commun..

[59] Buist G, Steen A, Kok J, Kuipers OP (2008). LysM, a widely distributed protein motif for binding to (peptido)glycans. Mol. Microbiol..

[60] Spiwok V (2017). CH/π interactions in carbohydrate recognition. Molecules.

[61] Hudson KL, Bartlett GJ, Diehl RC, Agirre J, Gallagher T, Kiessling LL (2015). Carbohydrate-aromatic interactions in proteins. J. Am. Chem. Soc..

[62] Houser J, Kozmon S, Mishra D, Hammerová Z, Wimmerová M, Koča J (2020). The CH-π interaction in protein-carbohydrate binding: bioinformatics and *in vitro* quantification. Chemistry-A European Journal..

[63] Keys AM, Kastner DW, Kiessling LL, Kulik HJ (2025). The energetic landscape of CH-π interactions in protein-carbohydrate binding. Chem. Sci..

[64] Machovič M, Svensson B, Ann MacGregor E, Janeček Š (2005). A new clan of CBM families based on bioinformatics of starch‐binding domains from families CBM20 and CBM21. FEBS J..

[65] García-Paz FdM, Del Moral S, Morales-Arrieta S, Ayala M, Treviño-Quintanilla LG, Olvera-Carranza C (2024). Multidomain chimeric enzymes as a promising alternative for biocatalysts improvement: a minireview. Mol. Biol. Rep..

[66] Kitago Y, Karita S, Watanabe N, Kamiya M, Aizawa T, Sakka K (2007). Crystal structure of Cel44A, a glycoside hydrolase family 44 endoglucanase from *Clostridium thermocellum*. J. Biol. Chem..

[67] Cicortas Gunnarsson L, Montanier C, Tunnicliffe RB, Williamson MP, Gilbert HJ, Nordberg Karlsson E (2007). Novel xylan-binding properties of an engineered family 4 carbohydrate-binding module. Biochem. J..

[68] Teh A-H, Sim P-F, Hisano T (2020). Structural basis for binding uronic acids by family 32 carbohydrate-binding modules. Biochem. Biophys. Res. Commun..

[69] Ficko-Blean E, Boraston AB (2006). The interaction of a carbohydrate-binding module from a *Clostridium perfringens* N-acetyl-β-hexosaminidase with its carbohydrate receptor. J. Biol. Chem..

[70] Røjel N, Kari J, Sørensen TH, Badino SF, Morth JP, Schaller K (2020). Substrate binding in the processive cellulase Cel7A: transition state of complexation and roles of conserved tryptophan residues. J. Biol. Chem..

[71] Taylor LE, Knott BC, Baker JO, Alahuhta PM, Hobdey SE, Linger JG (2018). Engineering enhanced cellobiohydrolase activity. Nat. Commun..

[72] Vermaas JV, Kont R, Beckham GT, Crowley MF, Gudmundsson M, Sandgren M (2019). The dissociation mechanism of processive cellulases. Proc. Natl. Acad. Sci. USA.

[73] Zajki-Zechmeister K, Kaira GS, Eibinger M, Seelich K, Nidetzky B (2021). Processive enzymes kept on a leash: how cellulase activity in multienzyme complexes directs nanoscale deconstruction of cellulose. ACS Catal..

[74] Chaudhari YB, Várnai A, Sørlie M, Horn SJ, Eijsink VG (2023). Engineering cellulases for conversion of lignocellulosic biomass. Protein Eng. Des. Sel..

[75] Wilkens C, Svensson B, Møller MS (2018). Functional roles of starch binding domains and surface binding sites in enzymes involved in starch biosynthesis. Front. Plant Sci..

[76] Baroroh U, Yusuf M, Rachman SD, Ishmayana S, Syamsunarno MRA, Levita J (2017). The importance of surface‐binding site towards starch‐adsorptivity level in α‐amylase: a review on structural point of view. Enzyme Res..

[77] Janeček Š, Mareček F, MacGregor EA, Svensson B (2019). Starch-binding domains as CBM families-history, occurrence, structure, function and evolution. Biotechnol. Adv..

[78] Ngo ST, Tran-Le PD, Ho GT, Le LQ, Bui LM, Vu BK (2019). Interaction of carbohydrate binding module 20 with starch substrates. RSC Adv..

[79] Zeng Y, Zheng H, Shen Y, Xu J, Tan M, Liu F (2019). Identification and analysis of binding residues in the CBM68 of pullulanase PulA from *Anoxybacillus* sp. LM18-11. J. Biosci. Bioeng..

[80] Kim SY, Kim H, Kim YJ, Jung DH, Seo DH, Jung JH (2021). Enzymatic analysis of truncation mutants of a type II pullulanase from *Bifidobacterium adolescentis* P2P3, a resistant starch-degrading gut bacterium. Int. J. Biol. Macromol..

[81] Kim EJ, Kim YJ, Yang SK, Seo YJ, Seo DH, Lim S (2024). Effects of carbohydrate binding module of pullulanase type I on the raw starch rearrangement by enhancing the hydrolysis activity. Food Biosci..

[82] Sorimachi K, Le Gal-Coëffet MF, Williamson G, Archer DB, Williamson MP (1997). Solution structure of the granular starch binding domain of *Aspergillus niger* glucoamylase bound to β-cyclodextrin. Structure.

[83] Koropatkin NM, Cameron EA, Martens EC (2012). How glycan metabolism shapes the human gut microbiota. Nat. Rev. Microbiol..

[84] Geerlings SY, Kostopoulos I, De Vos WM, Belzer C (2018). *Akkermansia muciniphila* in the human gastrointestinal tract: when, where, and how?. Microorganisms.

[85] Martens EC, Koropatkin NM, Smith TJ, Gordon JI (2009). Complex glycan catabolism by the human gut microbiota: the *Bacteroidetes* Sus-like paradigm. J. Biol. Chem..

[86] Cerqueira FM, Photenhauer AL, Doden HL, Brown AN, Abdel-Hamid AM, Moraïs S (2022). Sas20 is a highly flexible starch-binding protein in the *Ruminococcus bromii* cell-surface amylosome. J. Biol. Chem..

[87] Jiang H, Xie X, Ban X, Gu Z, Cheng L, Hong Y (2021). Flexible loop in carbohydrate-binding module 48 allosterically modulates substrate binding of the 1, 4-α-glucan branching enzyme. J. Agric. Food Chem..

[88] Photenhauer AL, Villafuerte-Vega RC, Cerqueira FM, Armbruster KM, Mareček F, Chen T (2024). The *Ruminococcus bromii* amylosome protein Sas6 binds single and double helical α-glucan structures in starch. Nat. Struct. Mol. Biol..

[89] Feng J, Qian Y, Zhou Z, Ertmer S, Vivas EI, Lan F (2022). Polysaccharide utilization loci in *Bacteroides* determine population fitness and community-level interactions. Cell Host Microbe.

[90] Mardo K, Visnapuu T, Vija H, Aasamets A, Viigand K, Alamäe T (2017). A highly active endo-levanase BT1760 of a dominant mammalian gut commensal *Bacteroides thetaiotaomicron* cleaves not only various bacterial levans, but also levan of timothy grass. PLoS One.

[91] Yin P, Du T, Yi S, Zhang C, Yu L, Tian F (2023). Response differences of gut microbiota in oligofructose and inulin are determined by the initial gut *Bacteroides*/*Bifidobacterium* ratios. Food Res. Int..

[92] Bondue P, Delcenserie V (2015). Genome of bifidobacteria and carbohydrate metabolism. Korean J. Food Sci. Anim. Resour..

[93] Moens F, Verce M, De Vuyst L (2017). Lactate-and acetate-based cross-feeding interactions between selected strains of lactobacilli, bifidobacteria and colon bacteria in the presence of inulin-type fructans. Int. J. Food Microbiol..

[94] Moens F, Weckx S, De Vuyst L (2016). Bifidobacterial inulin-type fructan degradation capacity determines cross-feeding interactions between bifidobacteria and *Faecalibacterium prausnitzii*. Int. J. Food Microbiol..

[95] Desai MS, Seekatz AM, Koropatkin NM, Kamada N, Hickey CA, Wolter M (2016). A dietary fiber-deprived gut microbiota degrades the colonic mucus barrier and enhances pathogen susceptibility. Cell.

[96] Ríos-Covián D, Ruas-Madiedo P, Margolles A, Gueimonde M, De Los Reyes-gavilán CG, Salazar N (2016). Intestinal short chain fatty acids and their link with diet and human health. Front. Microbiol..

[97] Silva YP, Bernardi A, Frozza RL (2020). The role of short-chain fatty acids from gut microbiota in gut-brain communication. Front. Endocrinol..

[98] Sokol H, Pigneur B, Watterlot L, Lakhdari O, Bermúdez-Humarán LG, Gratadoux JJ (2008). *Faecalibacterium prausnitzii* is an anti-inflammatory commensal bacterium identified by gut microbiota analysis of Crohn disease patients. Proc. Natl. Acad. Sci. USA.

[99] He X, Zhao S, Li Y (2021). *Faecalibacterium prausnitzii*: a next‐generation probiotic in gut disease improvement. Can. J. Infect. Dis. Med. Microbiol..

[100] Ghosh S, Whitley CS, Haribabu B, Jala VR (2021). Regulation of intestinal barrier function by microbial metabolites. Cell. Mol. Gastroenterol. Hepatol..

[101] Eilam O, Zarecki R, Oberhardt M, Ursell LK, Kupiec M, Knight R, *et al*. 2014. Glycan degradation (GlyDeR) analysis predicts mammalian gut microbiota abundance and host diet-specific adaptations. *mBio* **5:** 10.1128/mbio. 01526-01514. https://doi.org/10.1128/mbio.01526-14. 10.1128/mBio.01526-14 25118239 PMC4145686

[102] Jardon KM, Canfora EE, Goossens GH, Blaak EE (2022). Dietary macronutrients and the gut microbiome: a precision nutrition approach to improve cardiometabolic health. Gut.

[103] Zheng J, Huang L, Yi H, Yan Y, Zhang X, Akresi J, *et al*. 2024. Carbohydrate-active enzyme annotation in microbiomes using dbCAN. *bioRxiv.* 2024.2001. 2010.575125. https://doi.org/10.1101/2024.01.10.575125. 10.1101/2024.01.10.575125

