# Supplementary material for: Gatekeeping Dietary Fiber: The Role of Carbohydrate-Binding Modules in the Human Gut
Source: J Microbiol Biotechnol. 2026 Mar 16;36:e2601042. doi: 10.4014/jmb.2601.01042 (PMC13003330; doi:10.4014/jmb.2601.01042)
Supplement: Supplementary file 1 [file jmb-36-e2601042-supple.pdf]

J. Microbiol. Biotechnol. 2026. 36: e2601042

https://doi.org/10.4014/jmb.2601.01042

Supplementary Figure

Gatekeeping Dietary Fiber: The Role of Carbohydrate-Binding Modules in the Human Gut

Running Title: Gatekeeping Dietary Fiber via CBMs in the Human Gut

Inonge Noni Siziya[1], Cheon-Seok Park[2], and Dong-Hyun Jung[1]*

[1]*Division of Food and Nutrition, Chonnam National University, Gwangju 61186, Republic of Korea*

[2]*Department of Food Science and Biotechnology, Graduate School of Biotechnology and Institute of Life Science and Resources, Kyung Hee University, Yongin 17104, Republic of Korea*

***Corresponding author**: Dong-Hyun Jung

E-mail address: dhjung@jnu.ac.kr

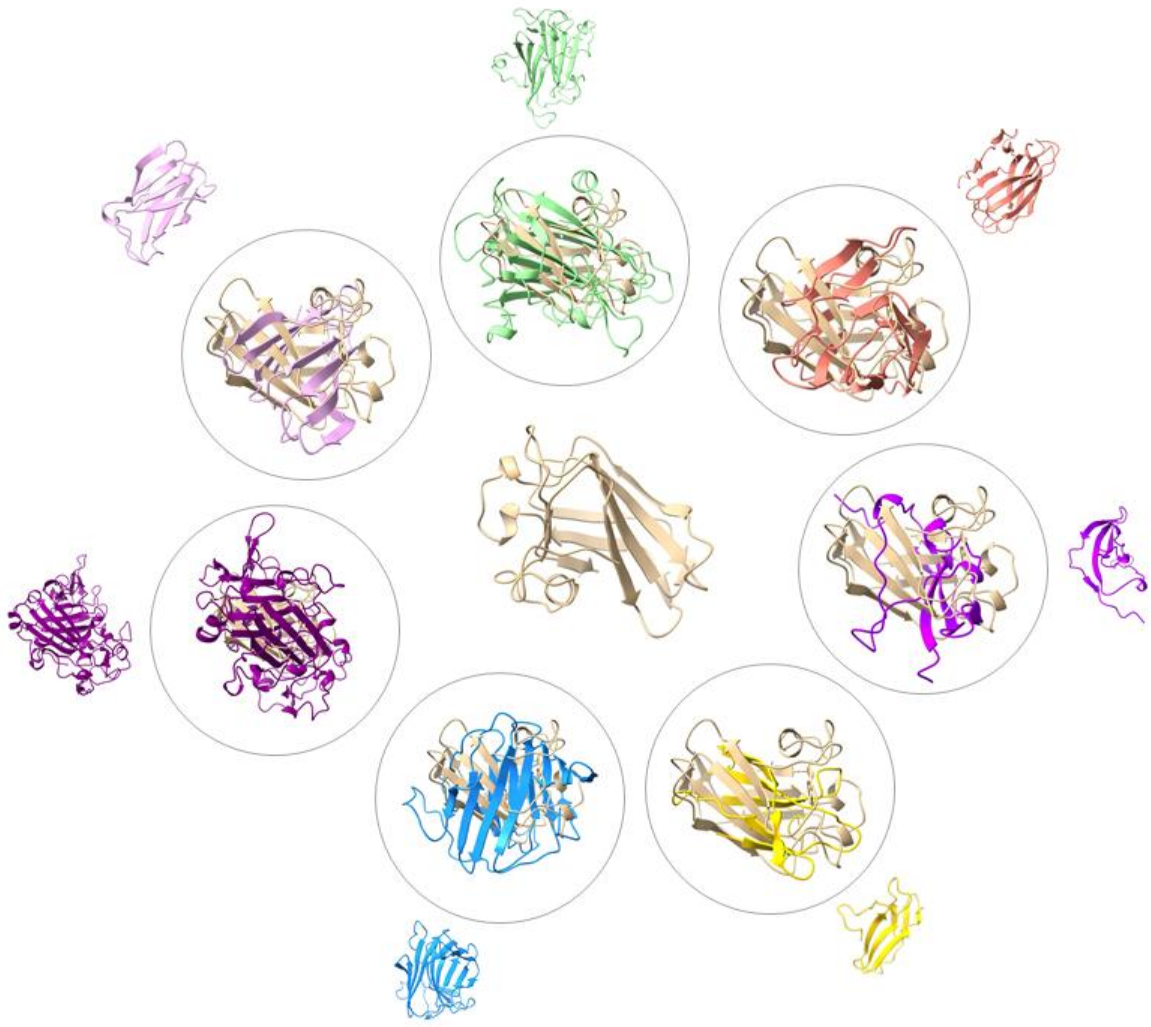

Fig. S1. Structural overlays of CBM32 with representative CBMs from distinct ecological guilds. CBM32 from *Bifidobacterium longum* (brown) is shown overlaid with representative carbohydrate-binding modules (CBMs) associated with different substrate-utilization pathways. Plant cell wall–associated CBMs include CBM2 from *Agathobacter rectalis* (pink) and CBM9 from *Bacteroides fragilis* (green). Recycling-associated CBM6 from *Bacteroides ovatus* is shown in peach. Resistant starch (RS)–associated CBMs include CBM20 from *Bacteroides thetaiotaomicron* (purple) and CBM25 from *Bifidobacterium breve* (yellow). Fructan-associated CBMs include CBM74 from *Ruminococcus bromii* (magenta) and CBM66 from *Anaerostipes hadrus* (blue). Despite low sequence similarity and divergent substrate preferences, overlays reveal conservation of the β-sandwich core architecture with variability concentrated in surface loops and binding faces. . Domain boundaries were determined using NCBI Conserved Domain Database (CDD) and InterPro. For each representative CBM family, the highest-confidence AlphaFold prediction (model_0) was used for structural comparison and visualization.
